# Isolation and identification of *Candida tropicalis* in sows with fatal infection: a case report

**DOI:** 10.1186/s12917-021-02821-0

**Published:** 2021-03-05

**Authors:** Lufeng Zhai, Ying Zhou, Yingxia Wu, Yunyun Jin, Qiaoyan Zhu, Shengguo Gao, Xuefeng Li, Zhe Sun, Yan Xiao, Baicheng Huang, Kegong Tian

**Affiliations:** National Research Center for Veterinary Medicine, No.3 Cuiwei Road, High-Tech District, 471003 Luoyang, Henan PR China

## Abstract

**Background:**

*Candida* is the common conditionally pathogenic fungus that infected human and animal clinically. *C. tropicalis* had been isolated from the skin and hair of healthy pigs, but with no report of fatal infection in gastrointestinal diseases.

**Case presentation:**

In a pig farm in Henan Province of China, about 20 % of pregnant and postpartum sows suffered from severe gastrointestinal diseases, with a mortality rate higher than 60 % in the diseased animals. The sows had gastrointestinal symptoms such as blood in stool and vomiting. Necropsy revealed obvious gastric ulcers, gastrointestinal perforation, and intestinal hemorrhage in the gastrointestinal tract, but no lesions in other organs. The microbial species in gastric samples collected from gastric ulcer of the diseased sows then was initially identified as *Candida* by using routine systems of microscopic examination, culture characteristics on the medium Sabouraud dextrose agar medium. The fungus was further identified as *C. tropicalis* by species-specific PCR and sequencing. This study revealed an infection of *C. tropicalis* in sows through gastrointestinal mucosa could cause fatal digestive system disease and septicemia.

**Conclusions:**

For the first time, a strain of *C. tropicalis* was isolated and identified from the gastric tissue of sows with severe gastrointestinal diseases. PCR and sequencing of ITS-rDNA combined with morphology and histopathological assay were reliable for the identification of *Candida* clinically.

## Background

*Candida* is the common conditionally pathogenic fungus that infected human and animal clinically by the species of *Candida albicans*, *Candida tropicalis*, *Candida glabrata*, *Candida parapsilosis*, and *Candida krusei *[1]. *Candida* can invade the skin, mucosa, and the internal organs, with two common syndromes of mucocutaneous candidiasis and invasive or deep organ candidiasis [2].

*C. tropicalis* is widespread in the environment, human skin, vagina, mouth, digestive tract, which would become pathogenic rapidly after alteration of the host immune system, causing the localized and even systemic infection [3, 4]. It was reported that in addition to human beings, *C. tropicalis* had been isolated from the skin and hair of healthy pigs, the feces of healthy poultry, the nasal cavity of healthy horses, the urine of dogs with cystitis and the external auditory canal of dogs and cats with otitis externa [5–8]. The infection of *C. albicans* is the main cause of gastrointestinal candidiasis in pigs. To date, there have been no report of lethal infection by *C. tropicalis* in pigs through digestive tract. In this study, the infection of *C. tropicalis* was identified from the gastric ulcer samples of sows with fatal gastrointestinal diseases.

## Case presentation

In late 2019, in a pig farm with a yearly scale of 5,000 sows and 10,000 fatting pigs located in Henan province, about 20 % of pregnant and postpartum sows were suffering from a serious digestive tract disease with a mortality rate higher than 60 %. While no abnormalities in the piglets from the sick sows could be found after being transferred to the healthy sows. The sick sows had gastrointestinal symptoms such as blood in stool and vomiting. Necropsy revealed obvious gastric ulcers, gastrointestinal perforation, and intestinal hemorrhage in the gastrointestinal tract, but no lesions in other organs. An automatic feeding system was adopted on the farm, and the granular feed of the sows was switch to the type of flake in one week before the sows showed clinical symptoms. The flake feed was sent for the tests of zearalenone, ochratoxin A, vomitoxin, T-2 toxin, and aflatoxin B1, B2, G1, G2, (Luoyang Sino-science Gene, China) after the disease occurred, and all the test results were negative.

**Fig. 1:**
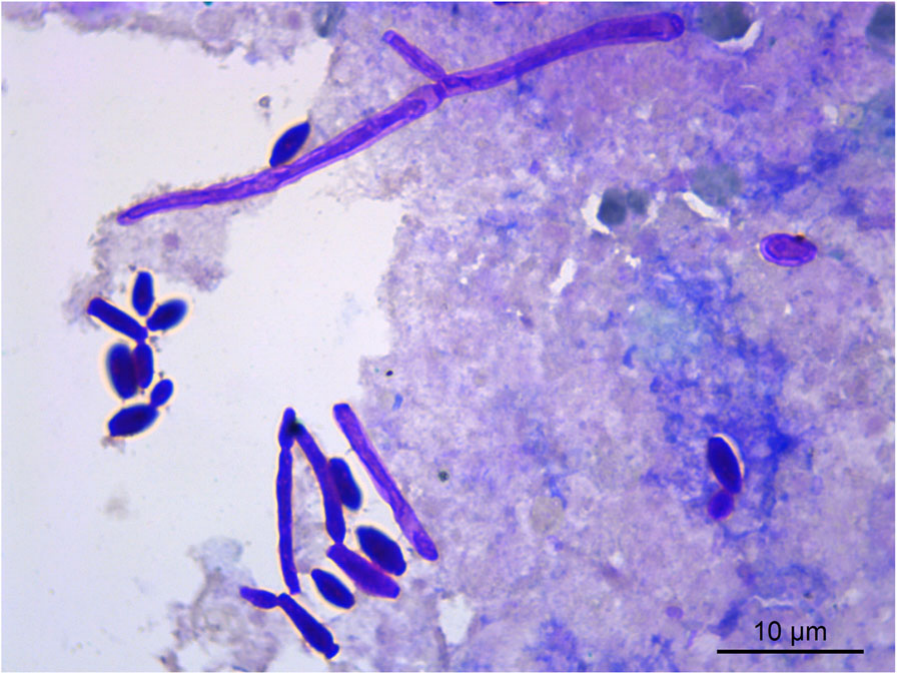
Morphology of yeast-like cells after PAS staining of gastric tissue (1000×)

**Fig. 2:**
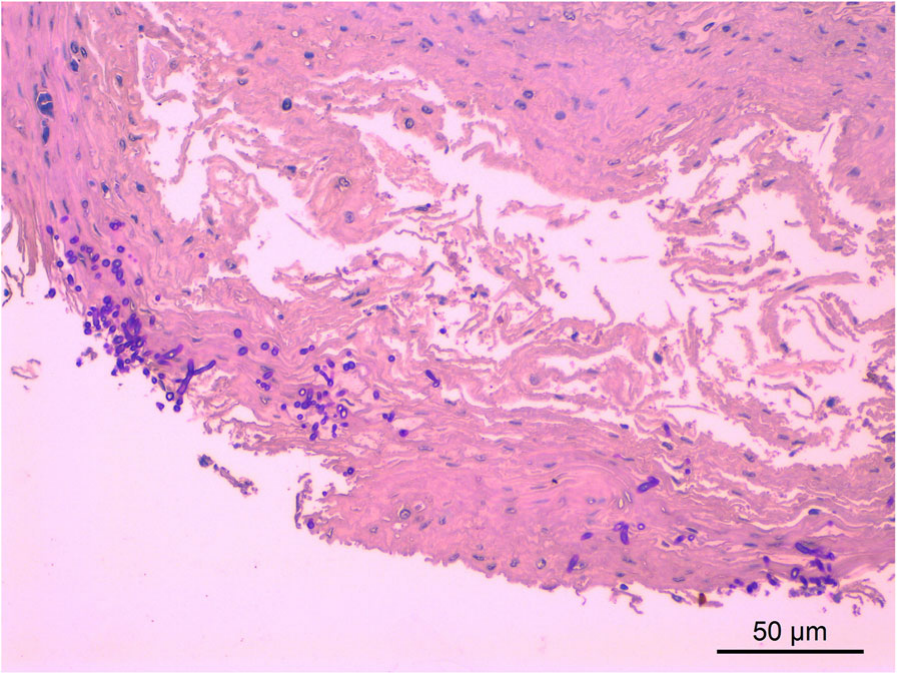
Histopathological observation of the gastric sample (H&E, 200×)

The damaged gastric mucosal tissues with ulcer of three dead sows were collected using sterile swabs from the sows dying from gastrointestinal diseases. The samples of gastric tissues were smeared on the clean glass slides. After fixation, the periodic acid–Schiff (PAS) staining kit (Solarbio, China) was used for morphological identification. The ovoid yeast-like cells that dark purple stained could be observed in gastric tissues of the three dead sows, with fungal spores and pseudohyphae (Fig. 1). In the histopathological assay, the gastric tissues were fixed in 10 % formalin fixative solution for 24 h, and then were placed in the embedded frame for dehydration and paraffin embedding. After slicing, staining, and mounting, the embedded samples were observed under the microscope. The results of H&E staining showed gastric mucosal epithelium injury and abscess, with yeast-like fungal spores and pseudohyphae in the exposed submucosa (Fig. 2).

**Fig. 3:**
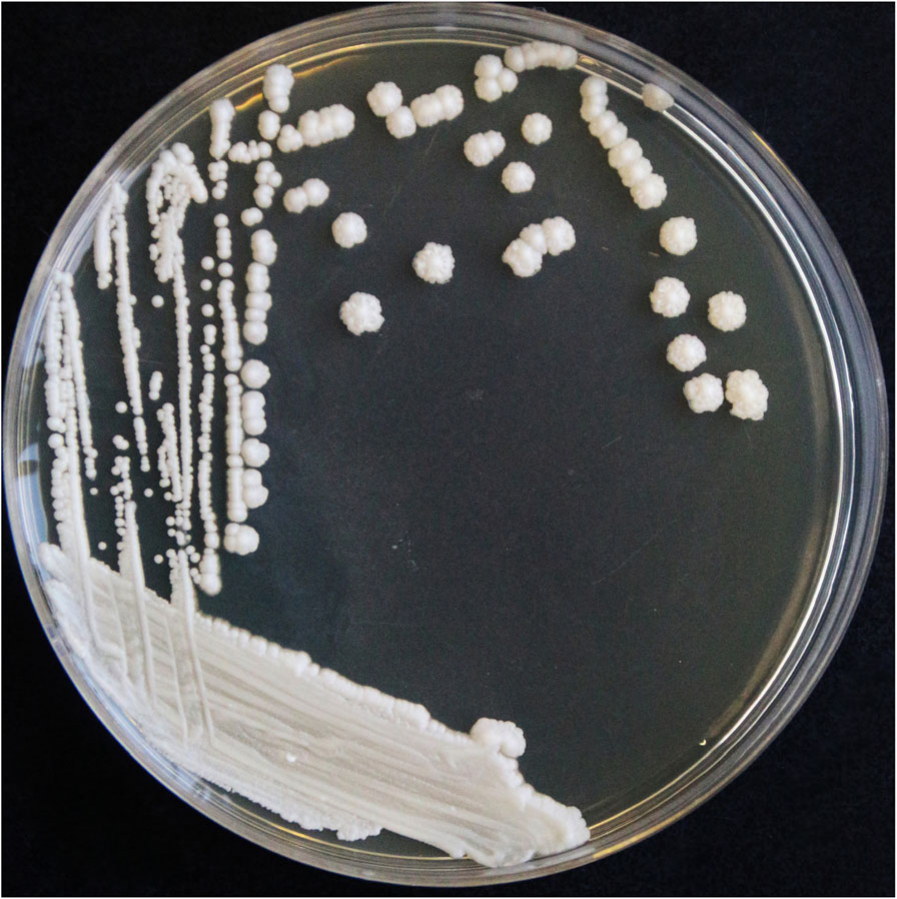
Growth of yeast-like colonies on SDA plate (48 h post incubation)

**Fig. 4:**
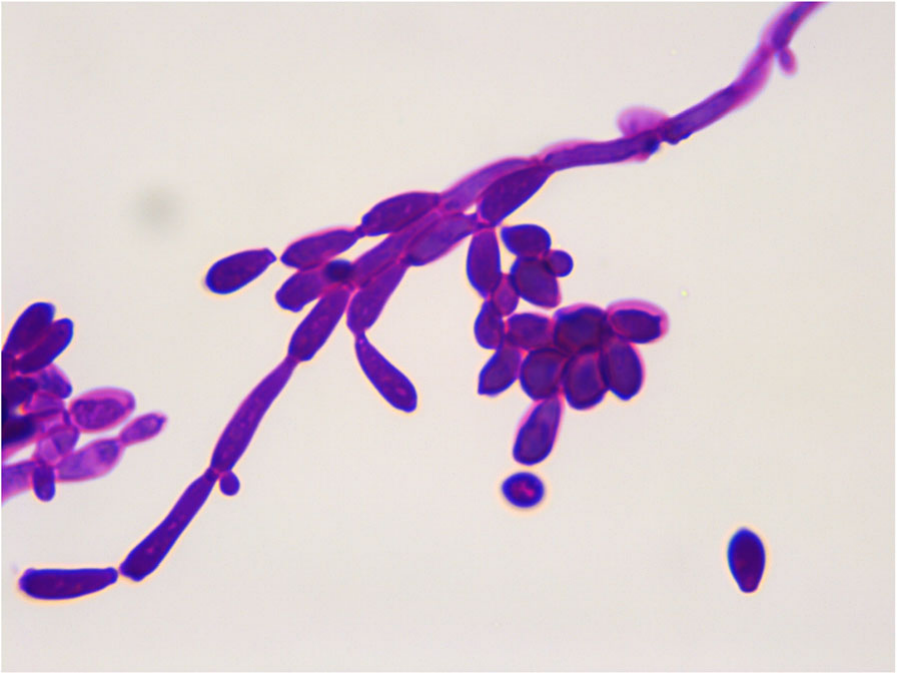
Morphology of yeast-like cells after PSA staining of the pure colony (1000×)

For culture characteristics assay, the samples of gastric tissues were inoculated on Sabouraud dextrose agar (SDA) plate at 28 ℃, and Tryptic soy agar(TSA) plate containing 10 % newborn bovine serum in 5 % CO_2_ at 37 ℃ for 3–5 days. After 48 h of incubation, a large number of monomorphic, cream-colored, smooth, glabrous colonies were observed on the SDA plate (Fig. 3), which consistent with the colony morphology of *Candida*, and no bacteria were grown on TSA plates. The *Candida*-like colonies on SDA plate were selected and streaked on SDA plate again for purification. After incubation at 28 ℃ for 3 days, one pure clone was picked for PAS staining and microscopy examination. After PAS staining, as observed in the smear microscopy, dark purple stained, ovoid yeast-like fungal spores and pseudohyphae of the pure colony could be found under microscopy (Fig. 4). The isolate was preliminarily identified as *Candida*.

**Fig. 5:**
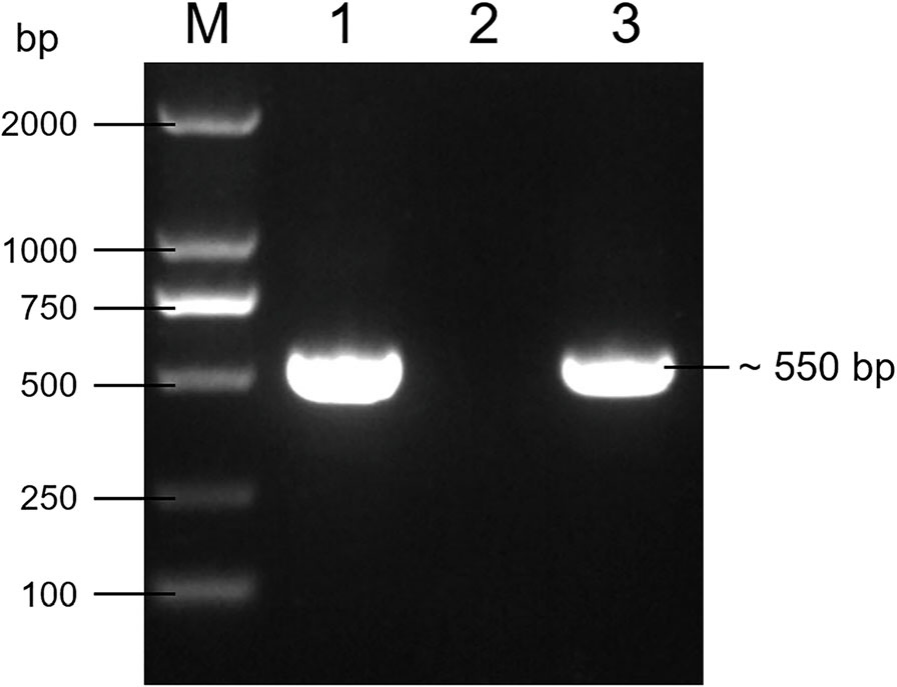
The result of PCR amplification of ITS-rDNA region [M: DL2000 DNA Marker; 1: the isolated strain; 2: Negative control (nuclease-free water); 3: Fungus positive control (*Candida Albicans* strain ATCC 10,231)]

The *Candida* isolate was further analyzed by PCR. DNA from the pure colony was extracted by fungi genomic DNA extraction kit (Solarbio, China) according to the manufacturer’s instruction. The internal transcribed spacers (ITS) region of rDNA (ITS-rDNA) were amplified using fungal universal primers Its1 (5’-TCCGTAGGTGAACCTGCGG-3’) and Its4 (5’-TCCTCCGCTTATTGATATGC-3’) [9]. The PCR cycle was as follows: 94 ℃ for 3 min, followed by 30 cycles at 94°C for 30 sec, 53 ℃ for 30 sec, 72°C for 1 min. and with a final extension at 72 ℃ for 10 min. The PCR products were analyzed by electrophoresis, an amplified fragment about 550 bp was obtained (Fig. 5).

To determine the species, the fragment about 550 bp identified by ITS PCR was sent for sequencing (GENEWIZ, China). The sequencing results were analyzed by BLAST on the National Center for Biotechnology Information (NCBI) database (). The sequence of ITS showed a nucleotide identity of 100 % compared with that of the *C. tropicalis* in GenBank (GenBank accession No.CP047875.1), confirming that the isolate was *C. tropicalis*.

## Discussion and conclusions

Usually, the causal factors for gastric ulcers in pigs including nutritional factors, physical aspects of feed [10], microbial infections, such as the bacteria of *Helicobacter suis* [11, 12]. In this case of sows with gastric ulcers, we confirmed that no nutritional, toxic of the feed or bacteria factors involved.

*C. albicans* is the main pathogen of human candidiasis. In recent years, the infection rate of non-Candida albicans, especially *C. tropicalis*, has increased [4, 13–17]. It has been reported that the candidiasis in swine is mainly caused by *C. albicans*. Here, for the first time, we reported the fatal infection of *C. tropicalis* in sows, and the *Candida spp.* was isolated from the gastric sample of infected animals.

*Candida* would rapidly proliferate at the damaged skin and mucosal surface, and then invade the body [4]. According to the investigation, the case in the pig farm may be related to feeding the sows with hard and flake feed after feed change, resulting the damage of mucosal ulcer in digestive tract, which promoted the infection of *C. tropicalis* by mucosal surface and caused fatal infection of the sows. Based on the laboratory diagnosis of *C. tropicalis* infection, the sows healthy in this pig farm was rapidly under controlled by strengthening the sanitation management, such as enclosing house, cleaning the feed trough, and feeding the digestible feed.

The traditional morphology and chromogenic medium-based methods for *Candida* identification have no high requirements for experimental conditions, while are susceptible to the influence of culture conditions and passage. The molecular biology method based on the genotype difference of species in the nucleotide sequences with high accuracy. The ITS-rDNA region of fungi has extensive sequence polymorphism, which is highly conserved among different strains within a species, but significantly different among different species within a genus. The PCR and sequencing analysis of ITS-rDNA sequences in different *Candida* species, including *C. albicans*, *C. parapsilosis*, *C. krusei*, *C. dubliniensis*, *C. guilliermondii* and *C. tropicalis*, were consistent with the results of morphological and biochemical characterization [18]. This feature makes ITS suitable for molecular identification of fungal species [19]. So, in this study, after smear microscopy and histopathological assay of the diseased sows’ samples, the case was preliminarily determined as *Candida* infection by the yeast-like fungi observation, and then further accurately and rapidly identified as *C. tropicalis* by ITS-rDNA PCR and sequencing.

## Data Availability

All data generated or analyzed during this study are included in this published article.
